# IMAGINE-ing interprofessional education: program evaluation of a novel inner city health educational experience

**Published:** 2017-02-24

**Authors:** Tina Hu, Kelly Anne Cox, Joyce Nyhof-Young

**Affiliations:** 1 Faculty of Medicine, University of Toronto, Ontario, Canada; 2 Office of Evaluations, Faculty of Medicine, University of Toronto, Ontario, Canada; 3 Centre for Ambulatory Care Education, Women’s College Hospital, Toronto, Ontario, Canada

## Abstract

**Background:**

Poverty is a key determinant of health that leads to poor health outcomes. Although most healthcare providers will work with patients experiencing poverty, surveys among healthcare students have reported a curriculum gap in this area. This study aims to introduce and evaluate a novel, student-run interprofessional inner city health educational program that combines both practical and didactic educational components.

**Methods:**

Students participating in the program answered pre- and post-program surveys. Wilcoxon signed-rank tests and descriptive thematic analysis were used for quantitative and qualitative data, respectively.

**Results:**

A total of 28 out of 35 participants responded (response rate: 80%). Student knowledge about issues facing underserved populations and resources for underserved populations significantly increased after program participation. Student comfort working with underserved populations also significantly increased after program participation. Valued program elements included workshops, shadowing, and a focus on marginalized populations.

**Conclusion:**

Interprofessional inner city health educational programs are beneficial for students to learn about poverty intervention and resources, and may represent a strategy to address a gap in the healthcare professional curriculum.

## Introduction

Socioeconomic status (SES) is a key determinant of health. Recent data have shown that one in seven or 4.8 million Canadians live in poverty.^1^ Low SES has been linked to other negative determinants of health, including: housing instability,^2^ non-adherence to medications due to inability to pay,^3^ food insecurity,^4^ delays and barriers to accessing care due to discrimination or lack of a fixed address,^5^,^6^ and greater disease severity.^3^ Compared to the general population, homeless people with low SES tend to have poorer health, experience a disproportionate burden of acute and chronic health conditions, have higher rates of mental health illnesses and substance abuse disorders,^7^,^8^ and significantly higher mortality rates.^9^–^11^

We believe that the majority of physicians and healthcare professionals will work with patients experiencing poverty and homelessness, regardless of their practice location or specialty and thus, healthcare students need to understand the impact of poverty on health status, know how to intervene to treat poverty, and directly train with vulnerable populations. However, surveys among healthcare students indicate a perceived curriculum gap in addressing poverty and providing resources for low income patients.^12^ Also, more evaluation research needs to be done on existing inner city health curricula for health profession students. Previous research has shown that incorporating inner city curricula positively influenced nursing students’ attitudes towards homeless populations^13^. Working interprofessionally at community-based organizations serving low-income or newcomer residents has been shown to improve communication skills among pre-clerkship students^14^ and help medical and pharmacy students develop skills to work more effectively with this population.^15^ Participating at a student-run free clinic serving marginalized populations has been shown to improve student knowledge and attitudes towards this population among medical students^16^ and enhance knowledge of interprofessional collaboration among physical therapy students.^17^

In response to feedback from health profession students at the University of Toronto indicating the need for more directed training when working with marginalized populations, two medical students (TH and KAC) developed an interprofessional inner city health experience program. The goals of the program were to: 1) enable students to gain knowledge on issues relevant to inner city populations; 2) practice their skills, such as interviewing patients and creating management plans, while shadowing at an interprofessional clinic serving primarily inner city populations; 3) develop an understanding of how to intervene and treat poverty and learn about available resources; and 4) learn about the role of other healthcare providers in poverty intervention. Table 1 presents the structure of the program.

The program had several components: 1) interactive workshops from guest speakers with lived experience and health practitioners, which focused on topics such as addictions, refugee health, non-medical interventions for poverty, and advocacy, and included interprofessional small group discussions; 2) clinical shadowing at the student-run IMAGINE clinic (Interprofessional Medical and Allied Groups for Improving Neighbourhood Environment) and other inner city clinics; and 3) a facilitated reflection session held in interprofessional small groups. A focus of the program was on interprofessional education: this was achieved through a variety of means such as facilitating case studies, workshop discussions, and reflection in interprofessional small groups as well as shadowing at an interprofessional clinic. At the clinic, a pair of students from different professions conducted the initial interview and examination with patients and brought this information to the larger interprofessional group of preceptors and students. A discussion was held with input from each profession to determine an effective management plan for each patient, which enabled students to learn about the scope of practice for each profession.

This study aimed to evaluate this student-run inner city health educational program among interprofessional healthcare students in order to improve the program for future students and add to the literature informing interprofessional training in inner city health care.

## Methods

This study was approved by the institutional ethics review board of the University of Toronto (Toronto, Ontario). Written informed consent was obtained from all students participating in the program. Participants were current students in medicine, social work, nursing, physician’s assistant, occupational therapy, physical therapy, speech-language pathology, pharmacy, and medical radiation sciences.

### Data collection and analysis

Participants were asked to complete pre- and post-program surveys. Each survey involved a quantitative Likert-scale portion (1 = least important, 5 = most important) and an open-ended response portion. The pre-program survey asked students about what skills and experiences they hoped to develop and their understanding of other health professions’ roles in treating poverty. The post-program survey asked about skill competencies developed during the program (derived from each health profession’s list of learning objectives), what students had learned about interprofessionalism, valuable aspects of the program, and program improvements.

All data were de-identified prior to analysis. Analysis of quantitative data was completed using Wilcoxon signed-rank tests with significance assigned at p < 0.05. Qualitative survey data were analyzed iteratively using descriptive thematic coding conducted by two separate reviewers using a consensus approach to resolve discrepencies.^18^

## Results

The overall cohort consisted of 35 participants (89% female), of whom 28 consented to survey completion (86% female, response rate of 80%). Participant average age was 24 ± 2.6 years. Disciplines represented were medicine (36%), nursing (14%), pharmacy (18%), social work (18%), and other professions (14%). About 52% of participants were in the first year of their programs, and 48% were in their second year. The proportion of students in their first year by discipline were: 42% (medicine), 100% (nursing), 40% (pharmacy), 40% (social work), and 67% (other professions).

### Skill competencies

Table 2 presents the competencies that participants hoped to gain during the program, in order of most personal importance, and associated rating using the Likert scale means. The top five desired skill competencies were to: 1) learn how to gather information and resources to develop a treatment plan, while considering the influence of factors such as social determinants of health; 2) develop therapeutic skills for effective client care; 3) identify social, physical, and economic determinants of health affecting the client and community; 4) understand how health promotion and protection strategies are applied in the community; and 5) develop assessment skills for effective client care.

Table 2 also shows the main skill competencies that students felt were addressed during their experience in the program, and their associated scores. The top five were: 1) identify social, physical, and economic determinants of health affecting the client and community; 2) reflect on your own performance, strengths, weaknesses, and personal development; 3) understand the roles and expertise of members within the interprofessional team; 4) develop skills for civic engagement related to health inequities; and 5) collaborate effectively within an interprofessional team.

### Program evaluation

Overall, the program was very well received by participants. Table 3 presents the post-program survey statements and their associated ratings. The workshops were seen as effective in enhancing students’ knowledge of issues facing inner city populations and how they affect health status. Participants also reported that the program effectively enabled them to learn about the role of other healthcare providers in poverty intervention through shadowing at the clinics and participating in interprofessional discussions about client management plans. Overall, the program stimulated their professional growth, and they would recommend the program to other students. Participants reported a significant increase in: 1) knowledge about issues facing underserved populations [median pre-program = 3.0 versus median post-program = 4.0, standard error (SE) = 24.03, z = 3.41, p = 0.001]; 2) knowledge of resources for underserved populations [2.0 versus 3.0, SE = 24.15, z = 3.69, p < 0.001]; and 3) comfort working with underserved populations [3.0 versus 4.0, SE = 17.81, z = 2.13, p = 0.03]. Participants did not report a significant increase in their interest in future work with underserved populations (p = 0.79).

### Student reflection

Students, asked to reflect on their personal reasons for joining this program, reported wishing to learn about and help underserved, marginalized populations and participate in an interprofessional team approach to providing healthcare to underserved populations. Four themes emerged from students’ written reflections:

#### Understanding marginalized populations

Many participants desired to learn about and help underserved and marginalized populations. Some had previous experience working with inner city populations, but most were neophytes. When asked why they joined this program, one participant answered: “to learn more about the less privileged communities and issues related to their access to healthcare.” Another was “interested in healthcare geared towards marginalized populations.”

#### Enhancing curriculum exposure

Several participants described not getting enough exposure to inner city health within their respective disciplines. One noted: “This is a great learning opportunity I don't think I will be able to gain solely from my schooling.”

#### Experience interprofessionalism

Interprofessionalism emerged as a major theme in participants’ reasons for joining the program. One participant described the program as “a unique opportunity to longitudinally work with students across several disciplines.” Another signed up “to witness and participate in an interdisciplinary team approach to providing healthcare to an underserved and often misunderstood population.”

#### Shadowing

Shadowing emerged as one of the most valuable program features. One participant wrote: “Shadowing a family physician who treated youth at a youth shelter. This was a valuable experience because I noticed many of the youth came in for surface issues such as a cold, but had many underlying mental health issues that couldn’t be addressed because there was no time.” Participants noted that shadowing would not have been as beneficial without the first day of workshops to orient them. As one participant noted: “I think one without the other really would have made the program incomplete.”

#### Workshops

Participants valued the educational aspect of the workshops. One student specifically highlighted the value of “hearing about the actual practical steps I could take to help future patients, for example, with income security.” Other valuable program elements included the “poverty tool”^19^ and “gain[ing] more information about the risk factors and issues.” Students gravitated toward practical tools and tips as translatable currency that they could carry forward in school and their future careers.

## Discussion

Similar to other community service learning programs,^15^ students were able to develop an understanding of and skills to work more effectively with clients from underserved settings. The overall program enabled participants to learn about the roles of other healthcare providers in poverty intervention through workshops, interprofessional small group case studies and discussions, and shadowing at an interprofessional clinic. The workshops were effective in enhancing respondents’ knowledge about issues facing inner city populations and provided them with practical tools and tips for their careers. Several skill competencies derived from learning objectives common to many healthcare professions were addressed in the program. Students highlighted the experience as a unique opportunity to work with other professions and learn about interprofessional collaboration while providing care to underserved populations. After program completion, participants felt that their knowledge about issues and resources for underserved populations and their comfort working with underserved populations were increased.

Given the significant number of people living in poverty and their unique health and social needs, it is important that medical and other health profession schools promote social responsibility by offering educational programs highlighting poverty intervention resources and enabling students to train directly with marginalized populations.^1^ Although medical students often start their medical training possessing abundant empathy and compassion, studies suggest that attitudes towards marginalized populations and empathy decline during their school experience.^16^,^20^–^22^ The “hidden curriculum” encompassing the implicit norms and values transmitted to students without being explicitly taught may play a role in influencing students’ declining empathy and attitudes towards certain populations.^16^,^23^,^24^ For example, although empathy and social responsibility are emphasized in the declared curriculum, students often witness behaviours in their day-to-day experiences that model self-interest, emotional detachment, and cynicism.^22^,^25^ Interventions such as service-learning opportunities working with underserved populations in the early stages of training have been proposed to counter the effects of the hidden curriculum.^16^,^22^ The inner city health experience represents a potential aid in preventing this decline in interest and empathy towards disadvantaged populations.

Study limitations include a relatively small sample size, as this was the first program iteration. However, our data suggest that students found that training with other professions in a program focused on inner city health was beneficial for professional development. This interprofessional inner city health educational program appears to be a worthwhile strategy to address a gap in the health profession curriculum. Areas for future research include long-term outcomes from program participation such as impact on career choice.

## Figures and Tables

**Table 1:** Program structure

Curriculum Development	Activities
Interactive Workshops (8 hours)	Large group sessions with interprofessional small groups for discussion
Clinical Shadowing (6–8 hours)	Interprofessional teams of students and preceptors from 5 disciplines (medicine, nursing, pharmacy, physical therapy, and social work) served clients experiencing homelessness or precarious status Students practiced skills such as interviewing and working in teams to create management plans
Facilitated Reflection Session (4 hours)	Held in interprofessional small groups Facilitators led the groups in case study discussion and reflection on their experiences

**Table 2:** Skill competencies students ranked in desirability pre-program and in attainment post-program

Skill Competencies	Skill competencies students hoped to develop during the program Likert Scale Means Pre-Programα	Skill competencies students developed during the program Likert Scale Means Post-Program*
Learn how to gather information and resources to develop a treatment plan, while considering the influence of factors such as social determinants of health	4.1	3.6 ± 1.0
Develop therapeutic skills for effective client care (example: communication skills, active listening, and counselling)	3.5	3.3 ± 1.0
Identify social, physical, and economic determinants of health affecting the client and community	3.1	4.2 ± 0.7
Understand how health promotion and protection strategies are applied in the community	3.0	3.8 ± 0.8
Develop assessment skills for effective client care (example: determining a differential diagnosis or management plan for clients)	2.9	3.3 ± 1.0
Understand the roles and expertise of members within the interprofessional team	2.8	3.7 ± 0.9
Develop skills for civic engagement related to health inequities	2.7	3.7 ± 0.9
Develop effective communication skills with clients	2.4	3.3 ± 1.0
Collaborate effectively within an interprofessional team	2.1	3.7 ± 0.9
Reflect on your own performance, strengths, weaknesses, and personal development	1.6	4.1 ± 0.7

α Participants ranked the top five skills/experiences they hoped to gain during the program (1 = least important, 5 = most important)

* Participants were asked how strongly they agreed the program enabled them to perform the listed competencies; responses could range from 1=strongly disagree to 5=strongly agree (n = 28).

**Table 3:** Post-program evaluation of the overall program

Statements	Likert Scale Means
Overall, my experience in this program was positiveα	4.6 ± 0.5
I would recommend this program to other studentsα	4.5 ± 0.7
How effective were the workshops in enhancing your knowledge about issues facing inner city populations and how they affect health status?*	4.4 ± 0.6
Participation in this program stimulated my professional growthα	4.4 ± 0.6
Overall, I am satisfied with my experience in this programα	4.4 ± 0.7
How effective was the program as a whole in enabling you to learn about the role of other healthcare providers in poverty intervention?*	4.0 ± 0.8
How effective were the workshops in developing your understanding of how to intervene and treat poverty?*	3.9 ± 0.8
How effective was the clinical shadowing in enhancing your knowledge about issues facing inner city populations and how they affect health status?*	3.4 ± 1.3

α where 1=strongly disagree and 5=strongly agree

* where 1=not at all effective and 5=extremely effective

## Appendix A Facilitated Reflection (Case Study and Reflection Questions)

### Case Study

Jorge is a 29 year-old man from El Salvador who was working as a seasonal temporary worker in southwestern Ontario for the summer. He decided to stay in Canada after having experienced a lot of discrimination in his hometown as a gay man. He has recently moved to Toronto and is couch surfing while he tries to find a good job and get his immigration status in order. A few times he has had to rely on shelters for a place to stay. He has no OHIP card and he shows up at the hospital after an accident he had at a construction site where he was working “under the table”. He fractured his vertebrae and will be unable to work his construction job for at least the next 6 months. When the care team meets with him, he acknowledges a lot of fear about how he’s going to afford to live and buy any medications you have prescribed to him. He tells you that he is quite depressed and has thought about ending his life several times.

What can you do to help him:

1. afford his medications
2. find sustainable housing
3. find a primary care provider who will look after him while he is uninsured
4. access any physio or other allied health care
5. find social support
6. find mental health support

### Facilitation questions

What do you think your health care profession struggles most with when it comes to addressing Jorge’s needs?

How would you imagine Jorge’s options would differ if he were a) living somewhere rurally instead of Toronto? b) a transwoman? c) a parent?

### Reflection Questions

#### What? (reporting what happened)

1. What were your hopes, fears, and expectations coming into this program?
2. Describe one event/interaction that sticks out to you the most [either during shadowing or workshops]. Who was there? What did you see and hear? What happened?

#### So what? (what did you learn?)

1. Why did that event stick out to you? Did it confirm or challenge previously held assumptions or stereotypes?
2. What did you learn from the event about: yourself, clients, inner city communities, interprofessional teamwork, and the role of healthcare providers and the healthcare system?
3. Why are social determinants of health and understanding poverty issues important to your profession?
4. Why are social determinants of health and understanding poverty issues important to interprofessional care as a team?
5. How would we truly approach advocacy and care of patients in poverty as an interprofessional team (i.e. We are working collaboratively to address patient priorities) instead of a multiprofessional one (we work in parallel with limited coordination and communication).
6. What was the value for you in learning in a group with other professionals?

#### Now what? (How will you think/act in the future as a result of this experience? Broader implications)

1. How has this experience caused you to reflect on your professional role with patients and on teams?
2. In reflecting on this session, what do you think may enable additional interprofessional education or learning about, from, and with each other?
3. What have you learned about this experience? How will you apply what you learned today in the future? Is there anything you want to learn more about?
4. Feedback for the program (what can be improved, strengths, etc).
